# Enhancing of Rabbit Sperm Cryopreservation with Antioxidants Mito-Tempo and Berberine

**DOI:** 10.3390/antiox13111360

**Published:** 2024-11-06

**Authors:** Lenka Kuželová, Andrea Svoradová, Andrej Baláži, Jaromír Vašíček, Vladimír Langraf, Adriana Kolesárová, Petr Sláma, Peter Chrenek

**Affiliations:** 1 Research Institute for Animal Production Nitra, National Agricultural and Food Centre (NPPC), Hlohovecká 2, 951 41 Lužianky, Slovakia; 2 AgroBioTech Research Centre, Slovak University of Agriculture in Nitra, Tr. A. Hlinku 2, 949 76 Nitra, Slovakia; 3 Institute of Biotechnology, Faculty of Biotechnology and Food Science, Slovak Agricultural University in Nitra, Tr. A. Hlinku 2, 949 76 Nitra, Slovakia; 4 Department of Zoology and Anthropology, Faculty of Natural Sciences, Constantine the Philosopher University in Nitra, Tr. A. Hlinku 1, 949 01 Nitra, Slovakia; 5 Institute of Applied Biology, Faculty of Biotechnology and Food Science, Slovak Agricultural University in Nitra, Tr. A. Hlinku 2, 949 76 Nitra, Slovakia; 6 Department of Animal Morphology, Physiology and Genetics, Faculty of AgriSciences, Mendel University in Brno, Zemedelska 1, 61300 Brno, Czech Republic

**Keywords:** rabbit, sperm cryopreservation, Mito-Tempo, berberine, sperm quality, oxidative stress

## Abstract

Cryopreservation plays a critical role in animal breeding and the conservation of endangered species, but it often compromises sperm characteristics such as morphology, motility, and viability due to oxidative stress. This study explores the antioxidative effect of Mito-Tempo (MT) and Berberine (BER) to enhance post-thaw sperm quality in rabbits. Pooled rabbit sperm samples were supplemented with different concentrations (0.0, 0.5, 5, 10, 50 µmol/L) of MT and BER. Sperm motility was evaluated using computer-assisted semen analysis, while viability, apoptosis, reactive oxygen species (ROS) levels, acrosome integrity, and mitochondrial function were assessed through flow cytometry. The results revealed that MT at 5 and 10 µmol/L and BER at 10 µmol/L significantly improved total and progressive motility, mitochondrial activity, and sperm viability compared to the control group. Furthermore, 10 µmol/L BER enhanced acrosome integrity, while both 5 µmol/L MT and 10 µmol/L BER effectively reduced ROS levels and apoptosis. This study is the first to demonstrate the protective effects of MT and BER on rabbit sperm during cryopreservation. By mitigating oxidative stress and reducing apoptosis, these antioxidants markedly improved post-thaw sperm quality, positioning MT and BER as promising agents for improving sperm cryosurvival.

## 1. Introduction

Rabbits hold significant importance in both the agricultural sector and biomedical research, making the preservation of valuable rabbit strains essential [1]. One effective method for maintaining these populations is through the cryopreservation of gametes. The cryopreservation of spermatozoa is a crucial technique for the ex situ conservation of genetic resources, enabling the preservation of genetic diversity and supporting reproduction in endangered species. This process, which involves freezing sperm cells at ultra-low temperatures, essentially halts biological activity, allowing genetic material to be stored for future controlled fertilization efforts [2]. Despite its long-standing use, cryopreserved semen generally yields lower fertility rates compared to fresh semen [3]. Cryodamage mechanisms include osmotic stress, cold shock, intracellular ice crystal formation, and excessive reactive oxygen species (ROS) production [4,5,6] along with disruptions in antioxidant defense systems [7,8]. Osmotic stress during cryopreservation is triggered by changes in cell volume due to water and solute movement across the sperm plasma membrane, leading to ROS generation [9]. Cold shock further intensifies oxidative stress (OS) and ROS production [10,11]. Recent studies indicate that ROS play a significant role in both reproductive physiology and pathology. At physiological levels, ROS are crucial for various processes related to sperm fertility, including proliferation, maturation, oocyte release, capacitation, hyperactivation, acrosomal reaction, and fertilization. However, excessive ROS production can lead to pathological responses, causing damage to cells and tissues [12,13,14]. During the freezing and thawing processes, the production of reactive oxygen species (ROS) and the disruption of oxidative metabolism significantly reduce sperm quality and viability [15]. Adding antioxidants and protective agents to the freezing media can help shield sperm from cryo-damage by neutralizing the harmful effects of ROS. These additives have demonstrated promising results in improving sperm preservation and viability during cryopreservation [15,16]. Plant-derived antioxidants are especially advantageous, as they contain a blend of naturally occurring, synergistic antioxidants and supporting compounds that help sustain antioxidant activity [17]. Numerous antioxidants have been proposed as beneficial in the treatment of male infertility to mitigate reactive oxygen species (ROS) and maintain sperm motility, viability, and functionality. These include compounds like vitamins E [18] and C [19], as well as selenium (Se) [20], zinc (Zn) [21,22], apigenin [23], or ellagic acid [24,25].

Mito-Tempo (MT) is a cell-permeable ROS scavenger that protects cells from oxidative stress by specifically targeting superoxide anions during the catalytic cycle [26,27]. It integrates the antioxidant piperidine nitroxide (TEMPO) with the lipophilic cation tri-phenylphosphonium (TPP+), enabling its accumulation in the mitochondria [28]. Adding MT to semen extenders has been shown to protect sperm quality in frozen-thawed human [29,30], buffalo [31], rooster [32], goat [33], bull [27], and cat epididymal spermatozoa [34]. Despite these promising findings, their specific impact on the cryopreservation of rabbit sperm remains unexplored.

Berberine (BER) is an alkaloid found in several plants, such as Oregon grape (*Berberis aquifolium*), turmeric (*Berberis aristata*), goldenseal (*Hydrastis canadensis*), and barberry (*Berberis vulgaris*) [35]. It exhibits a broad spectrum of pharmacological effects, such as antimicrobial, antioxidant, anti-inflammatory, cholesterol-lowering, and antidiabetic properties [36]. Species of the *Berberis* genus have demonstrated significant antioxidant activity in various assays, including 2,2-diphenyl-1-picrylhydrazyl (DPPH) scavenging, lipid peroxidation (LPO) prevention, reduction of oxyhemoglobin bleaching, and protection against DNA damage [37,38]. A study by Tvrdá et al. (2019) [39] demonstrated that supplementing bovine spermatozoa with berberine during both short- and long-term storage significantly improved sperm viability and motility while simultaneously reducing OS markers. To the best of our knowledge, there has been no prior research investigating the effects of BER on the cryopreservation of sperm in any species, including rabbits. Therefore, this study represents the first comprehensive examination of BER’s impact on rabbit sperm quality following freezing.

Additionally, as the effects of MT and BER on rabbit sperm characteristics post-cryopreservation have not been previously studied, our objective is to evaluate their potential protective benefits in this specific context. Furthermore, this research aims to identify the optimal concentration of each antioxidant to maximize the preservation and viability of rabbit sperm during cryopreservation.

## 2. Materials and Methods

### 2.1. Animals 

Sexually mature broiler rabbit males (n = 9) of M91 and P91 lines without overt evidence of genital tract infections were used in experiments. The rabbits were reared at the experimental rabbit farm SK U 18021 at the National Agricultural and Food Centre, Nitra (RIAP Nitra, Lužianky, Slovak Republic) and were housed in individual cages, fed with a commercial diet (KV; TEKRO Nitra, s.r.o., Nitra, Slovakia) and watered ad libitum. The photoperiod used was a ratio of 14 h light to 10 h dark. The temperature and humidity in the area were 17–20 °C and 60–65%, respectively.

### 2.2. Semen Processing and Experimental Design

Ejaculate was collected from each rabbit following a consistent schedule (twice a week for three consecutive weeks) using an artificial vagina. Fresh rabbit semen was immediately diluted with saline (0.9% NaCl; Braun, Melsungen, Germany) at a ratio of 1:10. The diluted semen was then assessed using a computer-assisted semen analysis (CASA) system with Sperm Vision™ 3.8 software (MiniTube, Tiefenbach, Germany) to evaluate motility parameters and concentration. Only semen samples meeting the following criteria were included in this study: greater than 70% progressive motility, more than 1.0 × 10^9^ spermatozoa/mL, and less than 10% abnormal sperm forms. Samples failing to meet these quality standards were excluded. All semen samples of good quality were pooled and cryopreserved using manual slow freezing procedure. Briefly, the pooled ejaculate was resuspended in a BotuCrio freezing medium (Nidacon, Mölndal, Sweden). Subsequently, ejaculate was divided into nine equal samples assigned to control group without antioxidants and the experimental groups contained 0.5, 5, 10 and 50 µmol/L MT (Sigma-Aldrich-SML0737, Sigma-Aldrich Corp., St. Louis, MO, USA)) or BER (Vitaberin-401/15, Vitaberin s.r.o., Bratislava, Slovakia). MT and BER were diluted to a 10 mM stock solution using dimethyl sulfoxide (DMSO) and stored at −20 °C until use. During the experiment, the appropriate doses were added to the BotuCrio extender to achieve the desired concentrations. The concentrations used in this study were established based on findings reported in previous publications across different species [29,32,39]. The samples were packaged in 0.25-mL straws (n = 4 per group; Minitüb, Tiefenbach, Germany), achieving a final sperm concentration of approximately 50 × 10^6^ spermatozoa per straw. The straws were immediately placed in a refrigerator (4–5 °C) for an equilibration period of 30 min. Following this, the straws were positioned 4 cm above liquid nitrogen (LN_2_) for 15 min (reaching temperatures between −125 to −130 °C) before being slowly submerged directly into LN_2_, where they were stored at −196 °C until analysis. For thawing, the straws were immersed in a temperature-controlled bath set to 37 °C for 30 s, after which their contents were transferred to an Eppendorf tube that had been pre-heated to 37 °C. The quality of both fresh and frozen-thawed spermatozoa was assessed using computer-assisted semen analysis (CASA) and flow cytometry, as outlined in the following sections.

### 2.3. Measurement of Semen Quality Parameters

#### 2.3.1. Reagents

All chemicals were obtained from ThermoFisher Scientific (Waltham, MA, USA), unless otherwise noted.

#### 2.3.2. Sperm Movement Characteristics

**Figure 1:**
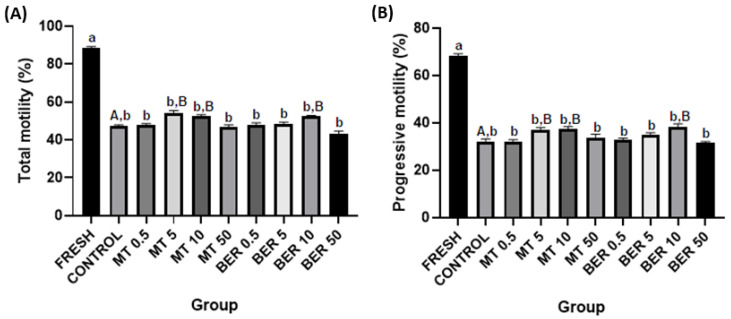
Effect of antioxidants MT and BER on rabbit sperm motility parameters after cryopreservation. (**A**) Total motility, (**B**) Progressive motility. Mean ± SD. Level of significance was set at 0.05. Columns labeled “a” and “b” indicate a statistically significant difference between the fresh sample and the thawed samples. Labels “A” and “B” represent a statistically significant difference between the control group without antioxidants and the experimental groups (*p * <  0.05, a vs. b; A vs. B).

Sperm motility and concentration were evaluated using a computer-assisted sperm analysis (CASA) system equipped with Sperm Vision™ 3.8 software (MiniTube, Tiefenbach, Germany). A 10 μL aliquot from each group of frozen-thawed samples was placed into a Makler counting chamber (37 °C; Sefi Medical Instruments, Haifa, Israel) and analyzed using the Sperm Vision™ 3.8 software under an AxioScope A1 light microscope (Carl Zeiss Slovakia, Bratislava, Slovakia). The analysis captured sperm motility and concentration in seven microscopic fields at a rate of 60 frames per second, completed in under one minute. The samples were assessed for total motility (TM; the percentage of motile spermatozoa with motility >5 μm/s) and progressive motility (PM; the percentage of progressively motile spermatozoa with motility >20 μm/s, Figure 1).

#### 2.3.3. Flow Cytometry Analysis

Sperm samples (aliquots from each fresh pooled ejaculates and frozen-thawed groups) were diluted to the concentration of 1 × 10^6^ spermatozoa in a phosphate-buffered saline (PBS, Ca- and Mg-free; Biosera, Nuaille, France) and subsequently incubated with specific chemicals. These chemicals were selected to evaluate various physiological attributes of the cells, such as viability, reactive oxygen species (ROS) production, mitochondrial activity, apoptosis, and acrosomal integrity, as previously detailed [40]. Samples were analyzed immediately after staining using a FACSCalibur flow cytometer (BD Biosciences, San Jose, CA, USA) equipped with 488 nm and 635 nm lasers. Fluorescent signals were captured using Cell Quest Pro™ software (BD Biosciences, San Jose, CA, USA), and each sample was analyzed for a minimum of 10,000 spermatozoa events. Flow cytometric data were processed with FlowJo™ v10.8.1 Software.

##### Sperm Viability Assessment

**Figure 2:**
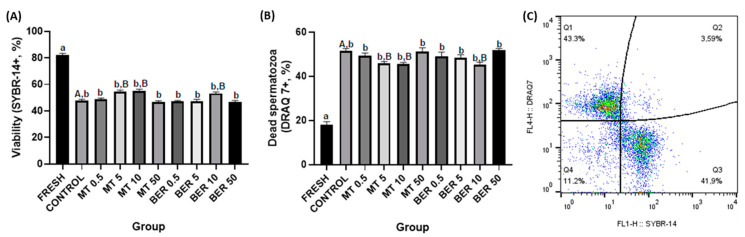
(**A**) Effect of antioxidants MT and BER on rabbit sperm viability after cryopreservation. (**B**) Effect of antioxidants MT and BER on the incidence of dead rabbit sperm after cryopreservation. Mean ± SD. Level of significance was set at 0.05 Columns labeled “a” and “b” indicate a statistically significant difference between the fresh sample and the thawed samples. Labels “A” and “B” represent a statistically significant difference between the control group without antioxidants and the experimental groups (*p * <  0.05, a vs. b; A vs. B). (**C**) Representative flow cytometry plots showing viable (Q3 quadrant) and dead spermatozoa (Q1 and Q2 quadrants).

The viability of spermatozoa was assessed using SYBR-14, a green fluorescent dye that permeates cell membranes (LIVE/DEAD^®^ Sperm Viability Kit, Thermo Fisher Scientific, Waltham, MA, USA), and DRAQ7, a far-red fluorescent nucleic acid dye (BioStatus Limited, Shepshed, UK) that stains the nuclei of dead or membrane-compromised cells. One million spermatozoa were incubated with 2.5 μL of SYBR-14 for 10 min at 37 °C, followed by co-staining with 3 μM DRAQ7 for another 10 min. Viable spermatozoa were identified as SYBR-14+ and DRAQ7-, while non-viable spermatozoa were classified as SYBR-14+/DRAQ7+ or as SYBR-14-/DRAQ7+ (Figure 2C).

##### Oxidative Stress Measurement

**Figure 3:**
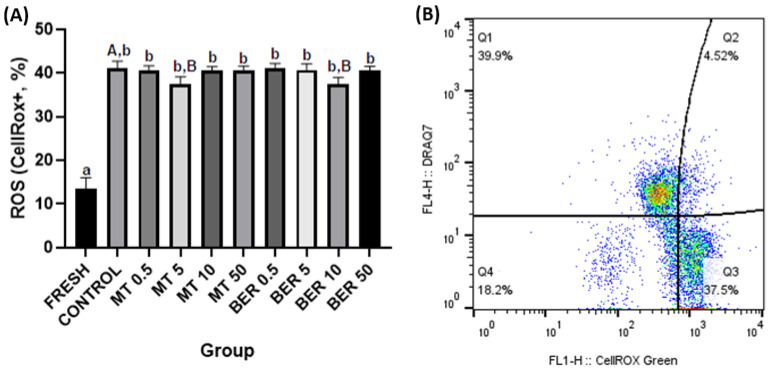
(**A**) Effect of antioxidants MT and BER on the rabbit sperm ROS generation after cryopreservation. Mean ± SD. Level of significance was set at 0.05. Columns labeled “a” and “b” indicate a statistically significant difference between the fresh sample and the thawed samples. Labels “A” and “B” represent a statistically significant difference between the control group without antioxidants and the experimental groups (*p * <  0.05, a vs. b; A vs. B). (**B**) Representative flow cytometry plots showing viable ROS positive spermatozoa (Q2 and Q3 quadrants).

Intracellular ROS levels were assessed using the CellRox Green assay (Thermo Fisher Scientific, Waltham, MA, USA). A semen sample (1 × 10^6^ spermatozoa) was incubated with CellRox (2.5 μM) for 30 min at 37 °C, followed by DRAQ7 staining. The percentage of ROS-positive spermatozoa was calculated (Figure 3B).

##### Mitochondrial Activity Evaluation

**Figure 4:**
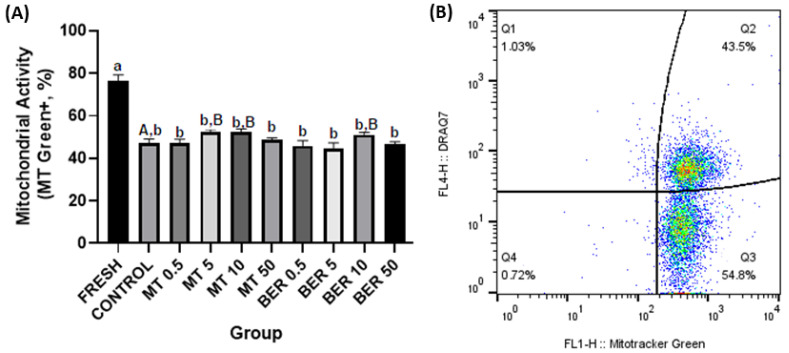
(**A**) Effect of antioxidants MT and BER on rabbit sperm mitochondrial activity after cryopreservation. Mean ± SD. Level of significance was set at 0.05. Columns labeled “a” and “b” indicate a statistically significant difference between the fresh sample and the thawed samples. Labels “A” and “B” represent a statistically significant difference between the control group without antioxidants and the experimental groups (*p * <  0.05, a vs. b; A vs. B). (**B**) Representative flow cytometry plots showing live spermatozoa with high mitochondrial activity (Q3 quadrant).

Mitochondrial activity was measured using MitoTracker^®^ Green FM (300 nM, MT Green, Thermo Fisher Scientific, Waltham, MA, USA). A semen sample was incubated at 37 °C for 10 min, and then centrifuged and stained with DRAQ7. The proportion of spermatozoa positive for MitoTracker (MT Green^+^/DRAQ7^−^) was assessed (Figure 4B).

##### Acrosome Integrity Evaluation

**Figure 5:**
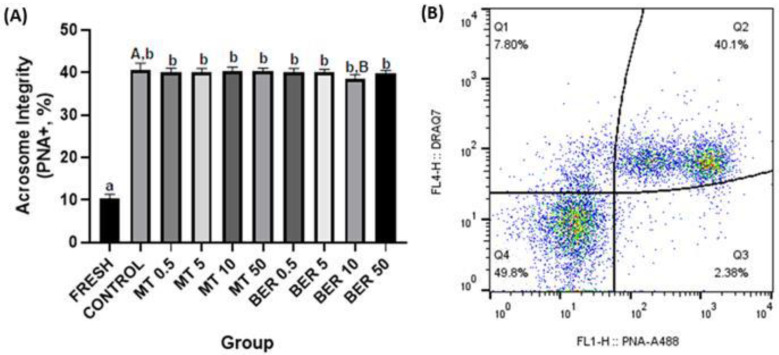
(**A**) Effect of antioxidants MT and BER on rabbit sperm acrosome integrity after cryopreservation. Mean ± SD. Level of significance was set at 0.05 Columns labeled “a” and “b” indicate a statistically significant difference between the fresh sample and the thawed samples. Labels “A” and “B” represent a statistically significant difference between the control group without antioxidants and the experimental groups (*p * <  0.05, a vs. b; A vs. B). (**B**) Representative flow cytometry plots showing PNA positive spermatozoa (Q2 and Q3 quadrants).

Acrosome integrity was assessed using peanut agglutinin (PNA, Thermo Fisher Scientific, Waltham, MA, USA) conjugated with Alexa Fluor 488 dye. One microliter of PNA solution was incubated with 1 × 10^6^ spermatozoa for 15 min in the dark. Following centrifugation and DRAQ7 staining, samples were analyzed to determine the percentage of acrosome-damaged spermatozoa (Figure 5B).

##### Apoptosis Assessment

**Figure 6:**
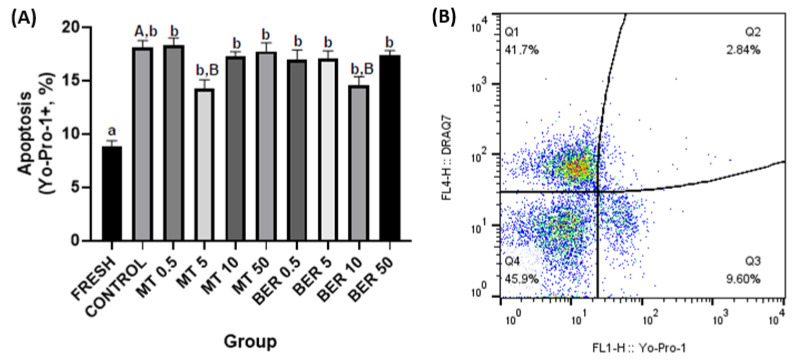
(**A**) Effect of antioxidants MT and BER on rabbit sperm apoptotic-like changes after cryopreservation. Mean ± SD. Level of significance was set at 0.05. Columns labeled “a” and “b” indicate a statistically significant difference between the fresh sample and the thawed samples. Labels “A” and “B” represent a statistically significant difference between the control group without antioxidants and the experimental groups (*p * <  0.05, a vs. b; A vs. B). (**B**) Representative flow cytometry plots showing Yo-Pro-1 positive spermatozoa (Q2 and Q3 quadrants).

**Figure 7:**
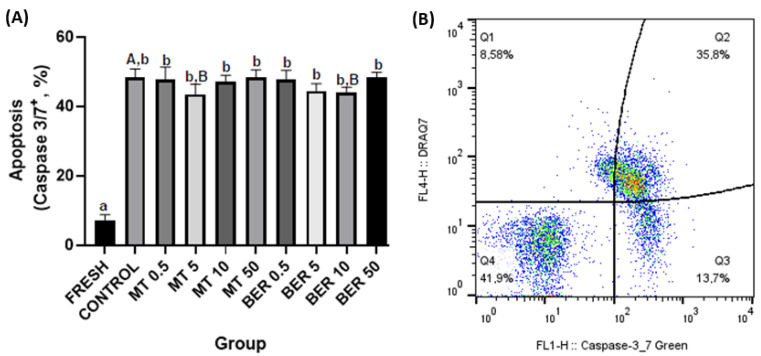
(**A**) Effect of antioxidants MT and BER on rabbit sperm apoptotic-like changes after cryopreservation. Mean ± SD. Level of significance was set at 0.05. Columns labeled “a” and “b” indicate a statistically significant difference between the fresh sample and the thawed samples. Labels “A” and “B” represent a statistically significant difference between the control group without antioxidants and the experimental groups (*p * <  0.05, a vs. b; A vs. B). (**B**) Representative flow cytometry plots showing Caspase 3/7 positive spermatozoa (Q2 and Q3 quadrants).

To determine apoptotic changes, spermatozoa were treated with Yo-Pro-1 (100 nM, Thermo Fisher Scientific, Waltham, MA, USA) and incubated for 15 min. After centrifugation, samples were stained with DRAQ7 and analyzed. Caspase activity was measured using the Caspase 3/7 reagent; (CellEventTM Caspase-3/7 Green Flow Cytometry Assay Kit, Thermo Fisher Scientific, Waltham, MA, USA). Caspase 3/7 specifically recognizes active caspase-3 and caspase-7 proteins [23]. Spermatozoa were incubated with the reagent and DRAQ7 before flow cytometric analysis. The proportion of Yo-Pro-1+ and Caspase 3/7+ spermatozoa was recorded (Figure 6B and Figure 7B, respectively).

### 2.4. Statistical Analyses

Experiments were repeated six times, and the Shapiro–Wilk test was used to evaluate normality. Data were analyzed using GraphPad Prism (version 9.3.1, GraphPad Software, San Diego, CA, USA) with two-way ANOVA and Sidak test for multiple comparisons. Results are expressed as mean ± SD, with statistical significance set at *p* < 0.05.

### 2.5. Use of Generative AI for Language Editing

The AI tool (OpenAI’s ChatGPT) was employed specifically for reviewing certain sections of the manuscript. This included grammar checks and overall language improvement to ensure the text met the publication standards. The AI-generated suggestions were carefully reviewed and incorporated as deemed appropriate by the authors.

## 3. Results

The quality parameters of the fresh pooled ejaculate showed high sperm motility and overall quality, which, as expected, significantly declined after thawing (Figures 1A–6A).

### 3.1. Effects of MT and BER on the Motility of Frozen-Thawed Sperm

CASA analyses revealed that concentrations of 5 and 10 μmol/L of MT and 10 μmol/L of BER significantly enhanced both total motility and progressive motility compared to the control group without antioxidants (*p*  <  0.05, Figure 1A,B).

### 3.2. Effects of MT and BER on the Viability of Frozen-Thawed Sperm

Flow cytometry analysis using SYBR-14 is presented in Figure 2A. The results showed that the percentage of viable sperm significantly increased with the supplementation of 5 and 10 μmol/L MT, reaching 54.28% and 54.89%, respectively, compared to 47.79% in the control group (*p*  <  0.05). Similarly, the addition of 10 μmol/L BER significantly boosted the viability to 53.02% compared to 47.79% in the control (*p * <  0.05). Correspondingly, the percentage of dead sperm in rabbit ejaculates after thawing was notably reduced with the addition of 5 and 10 μmol/L MT and 10 μmol/L BER, with percentages of 45.79%, 45.24%, and 46.71%, respectively, compared to 51.55% in the control group (*p * <  0.05, Figure 2B).

### 3.3. Effects of MT and BER on the ROS Generation of Frozen-Thawed Sperm

Flow cytometry revealed that supplementation with 5 μmol/L MT and 10 μmol/L BER led to significant reductions in sperm intracellular ROS levels, with percentages of 37.35% and 37.50%, respectively, compared to 41.13% in the control group (*p * <  0.05, Figure 3A).

### 3.4. Effects of MT and BER on the Mitochondrial Activity of Frozen-Thawed Sperm

As presented in Figure 4A, a significant increase of frozen-thawed rabbit sperm with high mitochondrial activity was recorded if treated with 5 and 10 μmol/L of MT and 10 μmol/L of BER, 52.38%, 51.65%, 50.48%, respectively vs. 47.24% (*p * <  0.05).

### 3.5. Effects of MT and BER on the Acrosome Integrity of Frozen-Thawed Sperm

MT supplementation did not result in significant improvements in sperm acrosome damage compared to the control across the tested doses. However, treatment with 10 µmol/L BER significantly reduced sperm acrosome damage to 38.28% in comparison to 42.62% of damage observed in the control group (*p*  <  0.05, Figure 5A).

### 3.6. Effects of MT and BER on the Apoptotic-like Changes of Frozen-Thawed Sperm

As shown in Figure 6A and Figure 7A, supplementation with 5 μmol/L MT and 10 μmol/L BER led to decreased expression of all apoptotic markers (Yo-Pro-1, and Caspase 3/7) compared to the control group (*p * <  0.05).

## 4. Discussion

This study aimed to explore strategies for improving rabbit sperm resilience against oxidative stress and enhancing the quality of sperm after thawing. Oxidative damage during cryopreservation is a significant factor that reduces the fertilization capacity of frozen-thawed sperm [41,42]. Sperm are particularly susceptible to ROS generation, especially during temperature fluctuations and cryo-injuries, due to their high content of polyunsaturated fatty acids, which makes their membranes prone to oxidative damage [43]. Elevated levels of ROS can trigger apoptosis, impair cellular metabolism, and disrupt the acrosome reaction in sperm [44]. Therefore, it is crucial to enhance freezing extenders, for example by incorporating potent antioxidants, to counteract the negative effects of ROS during cryopreservation. Researchers have extensively investigated the detrimental effects of increased OS caused by semen cryopreservation. Studies have shown that the incorporation of such antioxidants into cryopreservation media can significantly improve the success rates of sperm preservation across different species [11,45,46,47]. We selected the antioxidants MT and BER, which could potentially serve as cryoprotectants for rabbit semen. Our goal was to evaluate their protective effects during sperm freezing and determine the optimal concentration for their addition.

Sperm motility and kinetics are crucial for effective sperm transport to the fertilization site and are commonly used as key indicators of semen quality [48,49]. Mitochondria play a vital role in maintaining normal sperm function and energy balance through oxidative phosphorylation and ATP synthesis [50]. Cryogenic damage to mitochondria has been shown to negatively impact sperm motility by disrupting ATP transport processes [41]. In our experiment, we confirmed that the cryopreservation process significantly reduces sperm motility parameters. Concentrations of 5 and 10 μmol/L of MT significantly enhanced total motility and progressive motility compared to the control group without antioxidant supplementation. In our study, supplementation of the extender with 5 and 10 μmol/L MT significantly improved mitochondrial activity, and viability compared to the control group. The 5 μmol/L dose also substantially reduced ROS levels and apoptosis rates compared to the control. The addition of 10 μmol/L of BER also showed a similar positive significant effect on total and progressive motility. Furthermore, treatment with 10 µmol/L BER significantly reduced sperm acrosome damage, ROS production, and apoptosis compared to the control group. BER enhances mitochondrial activity and energy production in rabbit sperm, which are vital for sustaining sperm motility and overall functionality. This effect is particularly pronounced at a concentration of 10 µmol/L for rabbit sperm, as higher or lower doses did not demonstrate significant differences from the control group.

Previous studies showed that MT enhances motility and velocity in frozen–thawed sperm from human [29,30], rooster [32], or bull [27]. This aligns with our findings as both MT and BER demonstrated comparable effectiveness in improving sperm motility in cryopreserved samples, which is consistent with the study by Chen et al. [51], which examined the effects of various concentrations of berberine (10^−4^ to 10^−7^ mol/L) on human sperm motility. Their study analyzed semen samples from both normozoospermic and asthenozoospermic individuals, demonstrating that all tested concentrations of berberine effectively sustained both total and progressive sperm motility. Notably, motility increased even at a concentration of 10^−4^ mol/L, which corresponds to the 100 μmol/L concentration used in the current study. In contrast, our results showed that a concentration of 50 μmol/L did not yield any significant improvements in sperm behavior. This difference might stem from the fact that, in both studies, the sperm were stored rather than cryopreserved.

The freeze–thaw process places significant stress on sperm, leading to the overproduction of ROS, which causes DNA fragmentation, membrane damage, and cell apoptosis. Prolonged oxidative stress during cryopreservation is a major contributor to reduced sperm viability and fertility potential. Studies have reported significant benefits of using MT in protecting sperm DNA during the cryopreservation process. MT has been shown to reduce ROS levels and minimize apoptosis, improving post-thaw sperm quality and enhancing overall sperm function by mitigating oxidative damage [27,29,30]. In research on human sperm freezing, supplementation with MT was found to enhance sperm quality after thawing, improve antioxidant enzyme activity, and maintain mitochondrial membrane potential [29]. Zhang et al. [30] demonstrated that 10 and 100 μM concentrations of MT could alleviate cryodamage during freezing by modulating oxidative metabolism in spermatozoa from patients with compromised spermatogenesis. The activity of sperm mitochondria is closely linked to various quality characteristics of post-thawed spermatozoa. This finding is consistent with the current study, which showed that using optimal doses of MT resulted in enhanced mitochondrial activity and improved other quality parameters in sperm. Adding MT to the freezing media is believed to prevent mitochondrial Bax translocation, a process associated with mitochondrial dysfunction and apoptosis. Bax, a pro-apoptotic protein, translocates to the mitochondrial membrane under stress conditions like cryopreservation, where it promotes the release of cytochrome c, leading to cell death. By inhibiting this translocation, Mito-TEMPO helps maintain mitochondrial integrity and function [52,53,54]. Additionally, MT mitigates the excessive generation of oxygen-free radicals associated with sperm freezing–thawing through its hydroxylamine-like structure [27,30]. 

BER, as an alkaloid [55], primarily accumulates in mitochondria, where it plays a pivotal role in its intracellular distribution in mammalian cells [56]. This mitochondrial accumulation is driven by the mitochondrial membrane potential, which promotes the cellular entry of BER and inhibits its efflux from the cell. This rapid uptake by mitochondria not only helps maintain a concentration gradient across the cell membrane but also preserves the negative charge of the inner mitochondrial membrane [39,57]. BER may help protect mammalian cells from apoptosis or necrosis by preserving cell communication channels [58]. Studies have demonstrated that BER significantly improves sperm viability while reducing oxidative stress markers, following both short- and long-term storage of spermatozoa [39,51]. Moreover, BER has shown protective effects in cases of gossypol-induced testicular damage by improving semen quality, oxidative profile, and reducing inflammatory markers, further emphasizing its potential as an antioxidant in mitigating testicular and sperm damage [59]. These findings support BER’s role in enhancing semen quality during cryopreservation and in response to various stressors. BER enhances mitochondrial activity and energy production in sperm, which are vital for sustaining sperm motility and overall functionality. The antioxidant properties of 10 µmol/L BER may help inhibit lipid peroxidation of rabbit sperm plasma membranes and mitochondria, thereby protecting sperm integrity and function. Sperm plasma membrane and acrosome integrity are critical indicators of rabbit sperm quality, assessing the sperm’s ability to manage intracellular and extracellular components. Loss of membrane integrity leads to altered membrane permeability, cholesterol efflux, and calcium influx, which trigger capacitation-like changes and acrosomal damage, ultimately rendering sperm nonviable [60,61]. 

These findings demonstrate the importance of mitochondria-targeted antioxidants, such as MT and BER, in cryopreservation protocols. Both antioxidants significantly improved rabbit sperm motility, mitochondrial activity, and viability, particularly under cryogenic stress conditions. Our study’s findings on the selective effectiveness of different BER and MT concentrations in enhancing rabbit sperm quality and reducing oxidative stress contribute to a more nuanced understanding of optimal antioxidant use in cryopreservation. 

## 5. Conclusions

In conclusion, the addition of specific concentrations of antioxidants during rabbit semen cryopreservation proved beneficial, with 5 and 10 μmol/L for Mito-Tempo and 10 μmol/L for Berberine showing optimal effects. These antioxidants demonstrate strong potential as cryoprotectants for rabbit sperm, improving post-thaw viability and function. Future research should explore their mechanisms further and evaluate their broader application in cryopreservation protocols to enhance reproductive success across various species.

## Data Availability

The datasets during and/or analyzed during the current study are available from the corresponding author on reasonable request.
